# Monkeypox Epidemic: A Throwback From Smallpox Eradication

**DOI:** 10.7759/cureus.26577

**Published:** 2022-07-05

**Authors:** Lakshmi Deepak Bethineedi, Lakshmi Venkata Simhachalam Kutikuppala, Venkataramana Kandi

**Affiliations:** 1 General Medicine, Andhra Medical College, Visakhapatnam, IND; 2 Medicine, Konaseema Institute of Medical Sciences and Research Foundation (KIMS&RF), Amalapuram, IND; 3 Clinical Microbiology, Prathima Institute of Medical Sciences, Karimnagar, IND

**Keywords:** wild animals, humans, emergence, pandemic, monkeypox virus

## Abstract

The emergence of microbial diseases has become a major concern for humans. In the recent past, we have noticed the emergence and re-emergence of several microbes that include coronaviruses (severe acute respiratory syndrome coronavirus {SARS-CoV}, Middle-East respiratory syndrome coronavirus {MERS-CoV}, SARS-CoV-2), and others like Zika virus, Nipah virus, Influenza virus, and Ebola virus. These microbes have been noted to spill over from animals into humans. Several such microbes which were previously restricted to wild animals are now becoming responsible for infections in humans and have spread across the borders and resulted in pandemics. It has been more than two years since the discovery of the novel SARS-CoV-2 virus responsible for the coronavirus disease 2019 (COVID-19), and we are still struggling to cope-up with it and live normal lives. Recently, the monkeypox virus, which was confined to West and Central African countries, and caused endemic infections in monkeys and humans was associated with human infections in non-endemic regions like the United States of America (USA) and more than 30 other countries. Therefore, in this editorial, we attempt to put the spotlight on the monkeypox virus that is currently threatening to cause another widespread pandemic.

## Editorial

The poxviruses are a large group of deoxyribose nucleic acid (DNA) viruses that include several pox viruses which cause infections in humans and animals. The poxviruses belong to the family of Poxviridaeand are classified into two subfamilies based on their ability to infect vertebrates and non-vertebrates as Chondropoxvirinae and Entomopoxvirinae, respectively. The pox viruses that infect vertebrates including birds, animals, and humans are further classified into various genera that include Orthopoxvirus (animals and humans), Parapoxvirus (animals), Molluscipoxvirus (humans), Yatapoxvirus (animals and humans), Capripoxvirus (goats), Suipoxvirus (pigs), Leporipoxvirus (rabbits hares, squirrels), Avipoxvirus (birds), Crocodylidpoxvirus (crocodiles), and Cervidpoxvirus (deer and cattle). The variola virus (smallpox virus), belonging to the Orthopoxvirus group has been noted to frequently cause human infections. After the eradication of the smallpox virus, the vaccinia virus is maintained in the research laboratories and is used as a vaccine strain for immunization against the smallpox virus. Moreover, several pox viruses in animals have the potential to spill over and cause human infections, especially the cowpox virus, and the monkeypox virus [1].

**Figure 1:**
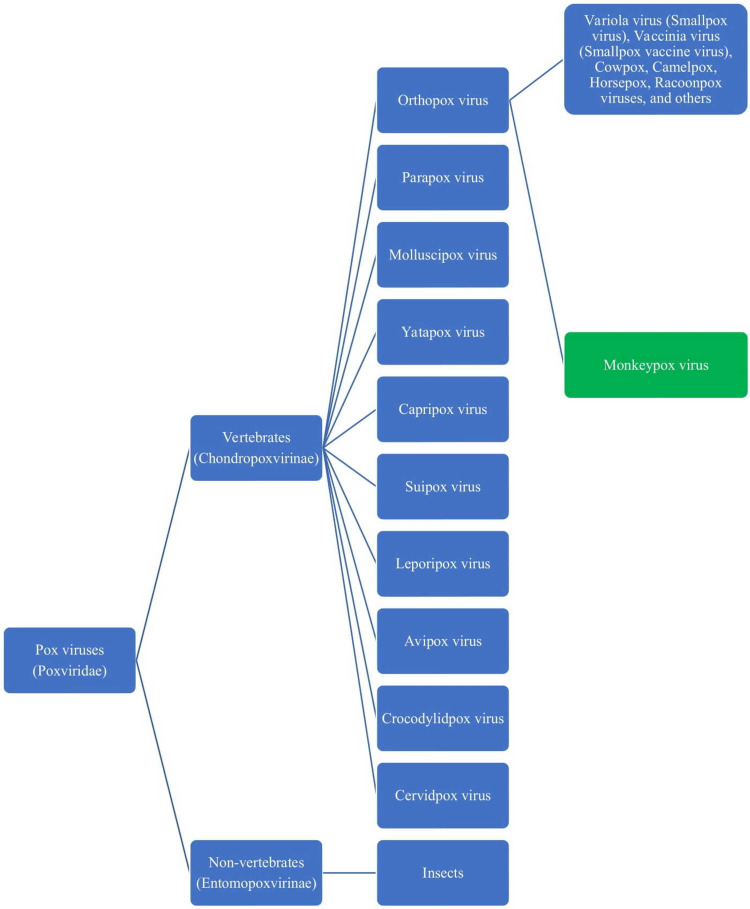
The classification of the pox viruses and the position of monkeypox virus

The cowpox viral infection among the milkmaids, and their immunity against severe infection with smallpox virus during the previous smallpox virus pandemics was responsible for the discovery of a vaccine against smallpox in humans by Edward Jenner, who was later called the father of immunization. The monkeypox virus, which belongs to the Poxviridae family, Chordopoxvirinae subfamily, and Orthopoxvirus genus, causes monkeypox disease, which is currently an infrequent zoonotic illness. The classification of the pox viruses and the position of the monkeypox virus are depicted in Figure 1.

Monkeypox and smallpox viruses are related closely and monkeypox symptoms are similar to smallpox. The similarity in presenting symptoms of fever and rash between the two viral infections poses an immense significance for effective management. Since the discovery of the monkeypox virus in 1970 in the Democratic Republic of the Congo (DRC), the monkeypox virus and its epidemiology remained unclear. Monkeypox infection spread through contact with infected vesicles and pustular fluids, dressings, and close contact. The contaminated surfaces and material need to be disinfected before further handling. Hand and surroundings hygiene, laundry of clothes, using personal protective equipment, and keeping a safe distance from patients might be useful in breaking the transmission chain [1]. Further randomized clinical trials will be beneficial to validate these observations. After the discovery in DRC, the monkeypox infection spread out to various other parts of the world such as Europe, Australia, America, UAE, and the Czech Republic. The exact causal link for widespread infection is yet to be studied. The most acceptable reason is the entry of new cases to non-exposed areas and further spread within them, but it has to be confirmed by studies.

The viruses that generally affected wild animals and were confined to wildlife have been able to successfully crossover into humans. Interestingly, these viral species have adapted extremely well to be able to establish themselves among humans thereby enabling human-to-human transmission. This has been the case with the novel Severe acute respiratory syndrome coronavirus-2 (SARS-CoV-2), the causative agent of COVID-19. The SARS-CoV-2 was linked to a wet market in Wuhan, China that was a place where different species of birds, seafood, and animals were sold. However, scientists have not been able to identify the exact animal/bird species from which the SARS-CoV-2 could have emerged [2].

Monkeypox is a viral disease that was noted to be restricted to monkeys living in the wild until human cases were identified. This was attributed to the uninhibited encroachment of the wildlife environment by humans, especially in the low-socioeconomic African countries for food and living. However, the virus has now been reported in non-African countries including the United States of America (USA), among others. Before the reports of monkeypox infections among people outside the African nations, the countries that were considered endemic for monkeypox included Cameroon, the Central African Republic, the DRC, and Nigeria [3].

The reason is the illegal transportation of wild, endangered, and exotic animals across the globe. The demand for such animals was due to their flesh, and medical benefits, among others. Moreover, several such animals have also gained demand as pets. Such human behavior could have facilitated the spillovers of several microbes that were restricted to animals into humans. The cause for concern here is the ability of the microbes to undergo genetic variations after the spillovers that help them to adjust to the new/human host. This has been proven by the previous emergence of microbes like the Ebola virus, human immunodeficiency virus (HIV), Zika virus, Nipah virus, severe acute respiratory syndrome coronavirus (SARS-CoV), SARS-CoV-2, and Middle-East respiratory syndrome (MERS) coronavirus, among others. Moreover, several such microbes that crossover from animals to humans and cause serious infections like the SARS-CoV-2 are complex and are not completely understood. Therefore, it is essential to investigate the exact origins of such microbes by extensively screening the wild, domestic, and pet animals throughout the world in a coordinated manner, similar to the one health initiative, that potentially enables scientists to predict any future emergences and the governments to implement control and prevention strategies [4].

Smallpox immunization with the vaccinia virus was found to be effective against monkeypox infections. However, routine immunization against smallpox is no longer recommended after the elimination of smallpox in 1980, and no orthopoxvirus vaccination program has been implemented in almost four decades. The laboratory and clinical confirmation of monkeypox viral infection in humans are complicated by the fact that this virus shares both pathological (eruptive lesions on skin and mucus membrane) and clinical characteristic features with other viruses including the Varicella-Zoster virus (chickenpox virus), and cowpox virus, among others.

Monkeypox infection can be diagnosed by isolating monkeypox DNA from a patient's sample and growing it in a viral culture or polymerase chain reaction (PCR). Alternatively, tests that indicate the presence of Orthopoxvirusin a patient specimen, barring patient exposure to another of the same genus, can be sufficient to diagnose, such as electron microscopy visualization, immunohistochemical staining for orthopoxvirus antigens, and serum studies for anti-orthopoxvirus IgM and IgG antibodies. The presence of IgM antibodies implies current or recent evidence of infection or exposure. The presence of IgG antibodies implies previous infection or immunity acquired passively through vaccination.

Medical treatment currently is at the clinical trials stage. From previous studies, it was noted that treatment with antiviral therapy consisting of tecovirimat (4-trifluoromethyl phenol derivative) was able to decrease the viral load by reducing the release of intracellular virus from the cell. Brincidofovir and cidofovir were found to inhibit viral DNA polymerase and reduce viral replication cascade [5]. But further studies are required to confirm the reliability of existing therapeutic interventions.

The eradication of the smallpox virus and discontinuation of smallpox vaccination could have contributed to a decline in herd immunity against monkeypox and resulted in the spread of the viral infection. However, the extensive endemic circulation of cowpox, and other vertebrate poxviruses among animals and people living in India and other geographical regions may confer protection by cross-immunity. Therefore, the development of effective vaccination and treatment strategies are to be dealt with in further studies to curb future epidemics. It is also important to perform genomic sequencing of the virus and identify the circulating strains/clades of the virus, their probable origins, host predilections, potential reservoirs, and the risk and severity of infections among humans.
